# Molecular characterization and distribution of *Schistosoma* cercariae collected from naturally infected bulinid snails in northern and central Côte d’Ivoire

**DOI:** 10.1186/s13071-019-3381-3

**Published:** 2019-03-19

**Authors:** Yves-Nathan T. Tian-Bi, Bonnie Webster, Cyrille K. Konan, Fiona Allan, Nana R. Diakité, Mamadou Ouattara, Diabaté Salia, Amani Koné, Adolphe K. Kakou, Muriel Rabone, Jean T. Coulibaly, Stefanie Knopp, Aboulaye Meïté, Jürg Utzinger, Eliézer K. N’Goran, David Rollinson

**Affiliations:** 1 Unité de Formation et de Recherche Biosciences, Université Félix Houphouët-Boigny, 22 BP 770, Abidjan 22, Côte d’Ivoire; 2 Centre Suisse de Recherches Scientifiques en Côte d’Ivoire, 01 BP 1303, Abidjan 01, Côte d’Ivoire; 3 Wolfson Wellcome Biomedical Laboratories, Department of Life Sciences, Natural History Museum, Cromwell Road, London, SW7 5BD UK; 4 Centre d’Entomologie Médicale et Vétérinaire, Université Alassane Ouattara de Bouaké, 27 BP 529, Abidjan 27, Côte d’Ivoire; 5 Institut National d’Hygiène Publique, Ministère de la Santé et de l’Hygiène Publique, Boulevard Du Port (Chu)-Treichville, Bp V 14, Abidjan, Côte d’Ivoire; 6 Swiss Tropical and Public Health Institute, P.O. Box, CH–4002, Basel, Switzerland; 7 University of Basel, P.O. Box, CH–4003, Basel, Switzerland; 8 Programme National de Lutte contre les Maladies Tropicales Négligées à Chimiothérapie Préventive (PNLMTN-CP), Ministère de la Santé et de l’Hygiène Publique, 06 BP 6394 Abidjan 06, Côte d’Ivoire

**Keywords:** *Bulinus forskalii*, *Bulinus globosus*, *Bulinus truncatus*, Côte d’Ivoire, Molecular markers, *Schistosoma bovis*, *Schistosoma haematobium*, Schistosome hybrids

## Abstract

**Background:**

Accurate identification of schistosome species infecting intermediate host snails is important for understanding parasite transmission, schistosomiasis control and elimination. Cercariae emerging from infected snails cannot be precisely identified morphologically to the species level. We used molecular tools to clarify the distribution of the *Schistosoma haematobium* group species infecting bulinid snails in a large part of Côte d’Ivoire and confirmed the presence of interspecific hybrid schistosomes.

**Methods:**

Between June 2016 and March 2017, *Bulinus* snails were sampled in 164 human-water contact sites from 22 villages of the northern and central parts of Côte d’Ivoire. Multi-locus genetic analysis (mitochondrial *cox*1 and nuclear ITS) was performed on individual schistosome cercariae shed from snails, in the morning and in the afternoon, for species and hybrid identification.

**Results:**

Overall, 1923 *Bulinus truncatus*, 255 *Bulinus globosus* and 1424 *Bulinus forskalii* were obtained. Among 2417 *Bulinus* screened, 25 specimens (18 *B. truncatus* and seven *B. globosus*) shed schistosomes, with up to 14% infection prevalence per site and time point. Globally, infection rates per time point ranged between 0.6 and 4%. *Schistosoma bovis*, *S. haematobium* and *S. bovis* × *S. haematobium* hybrids infected 0.5%, 0.2% and 0.4% of the snails screened, respectively. *Schistosoma bovis* and hybrids were more prevalent in *B. truncatus*, whereas *S. haematobium* and hybrid infections were more prevalent in *B. globosus*. *Schistosoma bovis*-infected *Bulinus* were predominantly found in northern sites, while *S. haematobium* and hybrid infected snails were mainly found in central parts of Côte d’Ivoire.

**Conclusions:**

The data highlight the necessity of using molecular tools to identify and understand which schistosome species are transmitted by specific intermediate host snails. The study deepens our understanding of the epidemiology and transmission dynamics of *S. haematobium* and *S. bovis* in Côte d’Ivoire and provides the first conclusive evidence for the transmission of *S. haematobium *× *S. bovis* hybrids in this West African country.

*Trial registration* ISRCTN, ISRCTN10926858. Registered 21 December 2016; retrospectively registered (see: http://www.isrctn.com/ISRCTN10926858)

**Electronic supplementary material:**

The online version of this article (10.1186/s13071-019-3381-3) contains supplementary material, which is available to authorized users.

## Background

Schistosomiasis, caused by dioecious digeneans of the genus *Schistosoma*, is a parasitic disease of considerable medical (~206.4 million people requiring treatment) and veterinary importance throughout tropical and subtropical regions [1]. There are 25 recognised *Schistosoma* species all of which have a two-host life-cycle with asexual reproduction occurring in a specific freshwater or amphibious snail and a sexual stage within the blood vessels of the definitive mammalian host [2]. Eggs are voided in the urine (*Schistosoma haematobium*) or faeces (most other species) of the infected definitive host. The separate sexes of the adult schistosomes enable interactions between male and female worms within their definitive hosts, while the asexual reproduction within the intermediate host snail gives rise to clonal larvae (cercariae) facilitating exposure and potential infection of definitive mammalian hosts that are in contact with the water [3, 4].

As obligate intermediate hosts, freshwater or amphibious snails constitute a crucial component for the transmission of *Schistosoma* species [5], with different snail species involved in the transmission of specific schistosome species or species groups. Different levels of snail-schistosome compatibility have been reported, depending on the species involved and this can be further complicated by geographical variation of both the snails and their schistosomes [5, 6]. Clarifying the interplay between these organisms, at a local/fine scale level, is not only vital for our understanding and monitoring of schistosome transmission but also for the application of snail control interventions to support schistosomiasis control and elimination [7–9].

Snail species of the genus *Bulinus* act as intermediate hosts for several schistosome species that belong to the *S. haematobium* species group in Africa and the Middle East [5, 6, 10, 11]. More precisely, *Bulinus truncatus* and *Bulinus globosus* have been shown to transmit both *S. haematobium* and *S. bovis*, which are responsible for human urogenital and livestock intestinal schistosomiasis, respectively. Additionally, *Bulinus forskalii* is an intermediate host for both *S. bovis* and the human rectal schistosomes *Schistosoma intercalatum* and *Schistosoma guineensis* [12]. Sympatric distribution of these snails occurs in many geographical and ecological settings resulting in co-endemic areas for multiple schistosome species [5, 13]. Moreover, the epidemiology of these schistosome species and their snail-schistosome relationships has been further complicated by interspecific hybridization between closely related schistosome species, such as *S. haematobium* and *S. bovis*, with hybridization now recognised to be commonly occurring in West Africa [14–16]. Hybrids can be identified using molecular techniques, which help to elucidate the epidemiology and potential impact on human and animal schistosomiasis. Worryingly, laboratory-bred hybrid schistosomes have been shown to exhibit vigour in terms of infectivity, fecundity, host (mammalian and snail) range and compatibility [14, 17, 18]. Moreover, Moné et al. [19] showed that the schistosomes responsible for the urogenital schistosomiasis outbreak in Corsica (Europe) were not just *S. haematobium* but also *S. haematobium* × *S. bovis* hybrids, with symptomatic and asymptomatic infections diagnosed in tourists. Along with the potential zoonotic transmission of livestock schistosomes, this issue could severely hamper control and elimination efforts. Cercariae emerging from infected snails cannot be precisely identified morphologically to the species level or as hybrids, and hence, molecular tools are required to characterize which species of snail is shedding which species or hybrid schistosome in different areas. Without such data, cercariae can be misidentified leading to incorrect epidemiological and transmission data [20].

In Côte d’Ivoire, it has been reported that *S. haematobium* is mainly transmitted by *B. truncatus* in the northern part of the country, while in central Côte d’Ivoire, both *B. truncatus* and *B. globosus* are involved in transmission [21–24]. In addition, the presence of *S. bovis* in northern Côte d’Ivoire has been highlighted with transmission involving three different snail species; *B. truncatus*, *B. globosus* and *B. forskalii* [23]. However, as there is migration of livestock from the north to the south [25] the occurrence of *S. bovis* is also expected in the central part of the country. Northern and central regions of Côte d’Ivoire are characterised by many small man-made dams built for crop irrigation, livestock watering and the development of sedentary cattle breeding [23, 26, 27]. The transmission of natural hybrids between *S. bovis* and *Schistosoma curassoni* (another cattle schistosome) has also been suggested to occur in Côte d’Ivoire [23] with evidence, based on cercarial behaviour, needing clarification with molecular methods.

Here we employed molecular tools to investigate the distribution and epidemiology of *S. haematobium* group species infecting *Bulinus* intermediate host snails in Côte d’Ivoire. Malacological surveys were conducted as part of a Schistosomiasis Consortium for Operational Research and Evaluation (SCORE) project, investigating the seasonal transmission of *S. haematobium* in a seasonal transmission setting in the northern and central parts of Côte d’Ivoire [28]. Schistosome cercariae, from infected *Bulinus* snails, were characterized using standard mitochondrial (*cox*1) and nuclear (ITS) genetic markers to determine species, and also to identify any inter-species interactions such as those observed in other West African countries [2]. The data are discussed in relation to (i) the distribution, abundance and seasonal changes of infected snails; (ii) the epidemiology of *S. haematobium* group species in Côte d’Ivoire, the host (*Bulinus*)-schistosome relationships and how this correlates to geography and season; and (iii) the complexities for the monitoring of human schistosomiasis transmission.

## Methods

### Study area

**Fig. 1:**
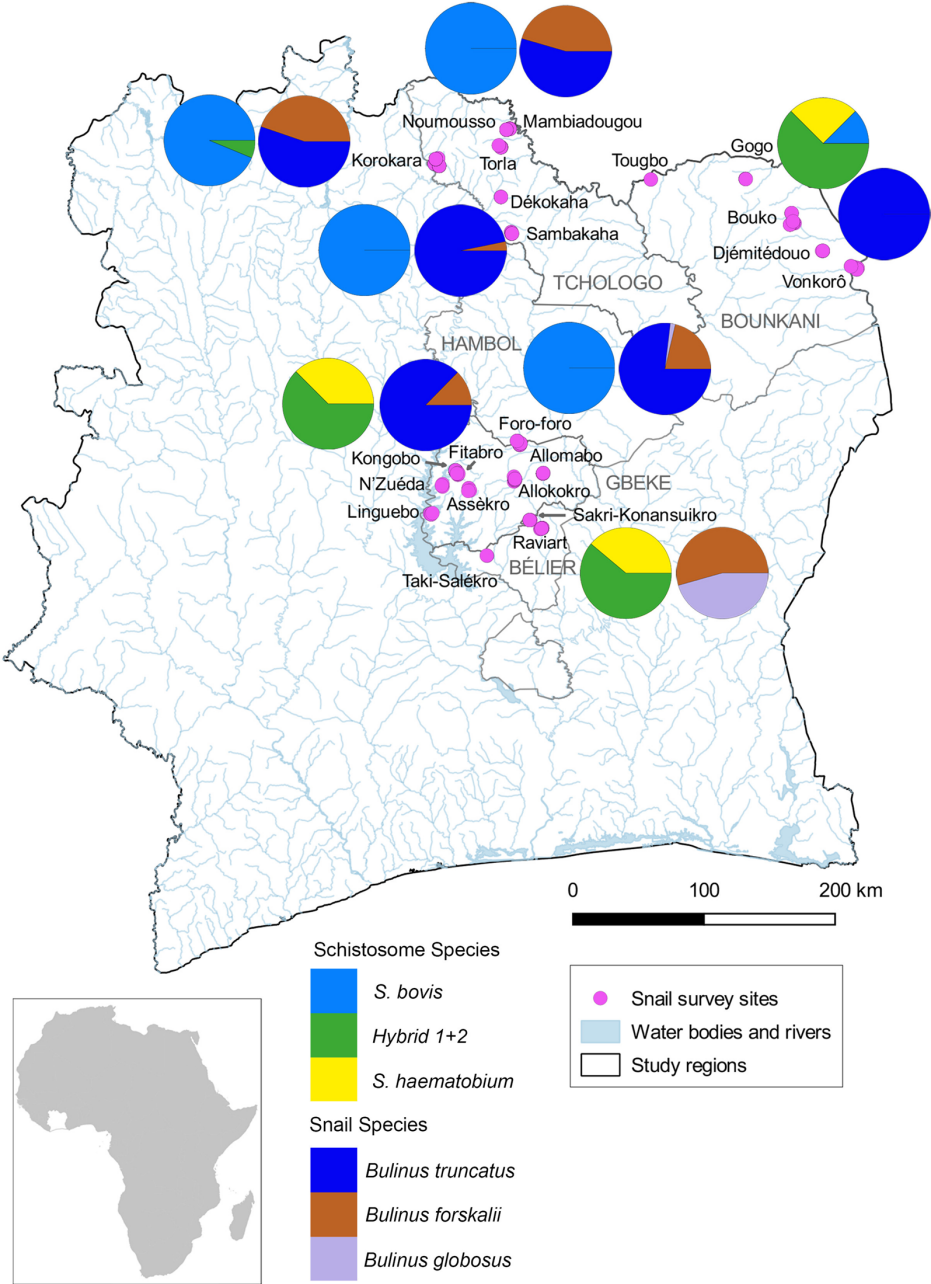
Map of Côte d’Ivoire showing the location of the 22 villages that were included in the study together with the distribution and proportions of the different *Bulinus* species found at the different survey sites where schistosome transmission was detected and the proportion of the different schistosomes being transmitted at the seven positive sites. The names of the villages are indicated in black font and names of regions are in grey. Data are merged for all three surveys conducted between June 2016-March 2017

As part of the SCORE project [28] in Côte d’Ivoire this study was carried out in the northern (regions of Tchologo and Bounkani) and central (regions of Hambol, Gkêkê and Bélier) areas that are endemic for schistosomiasis. Within these regions there are two distinct seasons; rainfall usually occurs between May and September with the remaining months being dry. There is a strong seasonal link between the presence of snails and the transmission of *S. haematobium* with infected snails generally found during the dry season (January and February) in man-made stagnant freshwater bodies [23]. As shown in Fig. 1, a total of 22 villages were surveyed; half of them in the north (Bouko, Dékokaha, Djémitédouo, Gogo, Korokara, Mambiadougou, Noumousso, Sambakaha, Torla, Tougbo and Vonkorô) and the other half in the central part of Côte d’Ivoire (Allokokro, Allomabo, Assèkro, Fitabro, Foro-Foro, Kongobo, Linguebo, N’Zuéda, Raviart, Sakri-Konansuikro and Taki-Salèkro).

### Snail surveys

In each study village, human-water contact sites were identified during a baseline malacological survey carried out in November 2015. Sites were georeferenced using a hand-held global positioning system (GPS; Garmin Sery GPS MAP 62, Olathe, KS, USA) device [28]. A total of 164 human-water contact sites were identified that were associated with dammed and natural lakes, irrigation canals (classified as main and secondary canals), rivulets and ponds (Additional file 1: Table S1). Snail surveys were carried out at each human-water contact site at three time points: June 2016, November-December 2016 and March 2017.

During each sampling session, snails were collected from each site by the same two investigators, using a long-handled scoop and/or forceps for a period of 15 min [13, 29, 30]. Snails were placed between two layers of moistened cotton in a Petri dish, labelled and transferred alive to a nearby laboratory. Snails were counted and morphologically identified using standard identification keys [5, 31]. Bulinid snails, specifically *B. truncatus* and *B. globosus*, were screened for infection by a standard cercarial emergence test. Additionally, *B. forskalii* were collected and recorded but, due to a high mortality rate post-capture, only those from the March 2017 surveys were screened for infection. All other snails were preserved in ethanol directly after collection.

### Testing snails for schistosome infections

Snails were kept in the laboratory, in batches of 15 individuals according to the sites where they were sampled, in transparent plastic tanks containing well water. Snails were maintained at a water temperature of 23–24 °C with a 12:12 hour photoperiod, and fed *ad libitum* with boiled lettuce and fish food. Water was changed at least twice per week. Snails were monitored for schistosome infection and survival/mortality using standard methods. Snails were checked for cercarial shedding on arrival and subsequently every other day over a period of one month. Cercarial shedding was stimulated by exposure of individual snails to artificial light at two time periods: between 10:00 and 12:00 h (morning) and between 13:00 and 15:00 h (afternoon). They were observed at two time periods to consider the different chronobiological/circadian rhythm of human (cercarial shedding more likely between 10:00–12:00 h) and livestock schistosomes (cercarial shedding more likely between 13.00–15.00 h) [32]. Snails were switched to fresh shedding containers between time periods and after the shedding period the containers were checked individually for the presence of cercariae under a binocular microscope [24]. For each snail, infection status (shedding of schistosome cercariae) was recorded, along with information on the site, collection date and whether cercarial shedding was observed in the morning or in the afternoon. Cercariae were morphologically identified as mammalian schistosomes based on keys produced by Frandsen & Christensen [33]. Only snails that shed *Schistosoma* cercariae were considered for subsequent molecular analyses.

### Preservation of schistosome cercariae and snails

Under a binocular microscope, individual cercariae from each snail were captured and pipetted individually onto Whatman FTA^®^ indicator cards (GE Healthcare Life Sciences; Amersham, UK) in a volume of 3 μl. Infected snails were individually preserved in ethanol for molecular identification. Samples were transported to the Natural History Museum, for molecular analyses.

### Molecular analysis of cercariae

#### DNA preparation

Using a Harris Micro-Punch^®^, a 2 mm disc was removed from the Whatman FTA^®^ indicator cards at the central point of where the sample was preserved. Up to four cercariae were punched from each infected snail and, whenever possible, this included two cercariae shed in the morning and two shed in the afternoon. The FTA punches were put in individual 0.2 ml tubes and the DNA from each cercaria was extracted using the alkaline elution method [34].

#### Molecular identification of schistosome cercariae

Multi-locus genetic analysis (mitochondrial *cox*1 and nuclear ITS) was performed on each individual cercaria for species and hybrid identification [15].

#### Nuclear ITS1 + 2 rDNA amplification

The complete ITS1 + 2 rDNA was amplified from each cercaria in 25 μl reactions containing 3 μl of template DNA, Illustra PuReTaq Ready-To-Go PCR Beads (GE Healthcare), 1 μl (10 pmol) of each forward (ITS2) and reverse (ITS1) primer [15, 35] and the PCR conditions described in Webster et al. [15].

#### Mitochondrial (mt) cox1 amplification

The diagnostic *S*. *haematobium* (*S*. *h*.)/*S*. *bovis* (*S*. *b*.) mtDNA *cox*1 marker was amplified from individual cercariae in 25 μl reactions containing 3 μl of each DNA sample, 1 μl (10 pmol) of each primer Asmit1 (forward), Sh.R (reverse) and Sb.R (reverse) [35] and the PCR conditions described in Webster et al. [15].

Positive controls of *S. haematobium* and *S. bovis* DNA (available from SCAN [36]) and a negative (no template DNA) control were used alongside each set of ITS and *cox*1 PCRs. Results were visualised by running 4 μl of each reaction on a 2% TAE gel stained with GelRed^TM^ (Biotium Inc, CA, USA).

#### Sequencing analysis

Positive PCR reactions were purified using QiaQuick PCR Purification Kit (Qiagen Inc., Santa Clarita, CA, USA) according to the manufacturer’s protocol and then Sanger sequenced, using a dilution (1 pmol) of the original PCR primers, on an Applied Biosystems 3730 automated sequencer (Applied Biosystems, Foster City, CA, USA). The sequences were assembled and edited manually using Sequencher version 4.5 (Gene Codes Corp., Ann Arbor, MI, USA). The identity of the *cox*1 and ITS sequences from the individual cercariae were confirmed by comparison to reference data [15] and also using the Basic Local Alignment Search Tool (BLAST). *Cox*1 and ITS genetic profiles were assigned to each individual cercaria to confirm species identity. *Cox*1 sequence data were submitted to the GenBank database.

### Snail molecular identification/confirmation

#### Sample preparation and DNA extraction

All snails found to be shedding schistosome cercariae were subjected to molecular identification to species level. Photographic images were taken of the snail shells before the soft tissue was placed in TE buffer with a pH 7.4 for 1 h in order to remove any remaining ethanol and to rehydrate the tissue. Total genomic DNA was isolated from the head/foot of each snail using DNeasy Blood and Tissue kit (Qiagen Inc., Santa Clarita, CA, USA) according to the manufacturer’s protocol with an overnight digest and the DNA was eluted in a total volume of 200 μl purified water.

#### Amplification of the partial cox1 mtDNA region

A PCR amplification of a partial cytochrome *c* oxidase subunit 1 (*cox*1) sequence was performed on each snail extract using Folmer primers (LCO1490 and HCO2198) [37] with the same PCR conditions as described in Kane et al. [38]. Amplicons were visualised on a 2% TAE GelRed agarose gel; positive reactions were purified and sequenced as described earlier. Snail species were confirmed by comparing the sequence data obtained from each individual to data available on GenBank.

### Statistical analyses of snail-schistosome combinations

The numbers of each snail species collected were computed and statistically analysed from each site and per survey date. Snail infection prevalence and corresponding 95% confidence intervals (CIs), based on Clopper-Pearson method [39], were calculated per type of water body for each village, for any schistosome infection and individual species. Different analysis parameters were included: time points, intermediate host snail species, time of cercarial emergence and geographical origin. Statistical analyses were based on multi-sample tests of proportions using the Fisher’s exact test in order to detect any significant differences. Statistical analyses were performed at the 5% significance level using the software STATA version 12.1 (StataCorp.; College Station, TX, USA). Mapping of snail and schistosome species was undertaken in Quantum GIS (QGIS) version 2.41.21 (Essen, Germany) and in R version 3.4.0 [40]. Water body data acquired from the DIVA-GIS database (http://www.diva-gis.org/) and administrative area shape file data from the Global Administrative Areas Database (GADM; https://gadm.org/).

## Results

The numbers of snails collected and those positive for schistosome infections at each time point and at each water contact site per village are given in a supplementary file (Additional file 1: Table S1). Snail species identification was based on morphology, except for the infected snails that were identified using molecular methods (Table 1).

**Table 1:** Molecular identification of each positive *Bulinus* snail and their schistosome cercariae, collected during the three malacological surveys carried out in the northern and central parts of Côte d’Ivoire

Time of survey	Village (part)	Snail species (no.)	Cercariae identification (no. of cercariae analysed)	
			Morning	Afternoon
June 2016	Noumousso (N)	*B. t.* (1)	*S*. *b.* (2)	*S*. *b.* (2)
	Foroforo (C)	*B. t.* (1)	*S*. *b.* (2)	*S*. *b.* (2)
	Kongobo (C)	*B. t.* (2)	Hybrid-1 (2)	Hybrid-1 (3)
November-December 2016	Korokara (N)	*B. t.* (2)	*S*. *b*. (3)	*S*. *b.* (3)
	Sambakaha (N)	*B. t.* (3)	*S*. *b.* (4)	*S*. *b.* (5)
	Raviart (C)	*B*. *g.* (1)	Hybrid-1 (1)	–
		*B*. *g.* (2)	*S*. *h.* (2)	*S*. *h.* (4)
		*B*. *g.* (1)	*S*. *h.* (1)	Hybrid-1 (2)
		*B*. *g.* (1)	Hybrid-1 (1)	Hybrid-1 (1)
		*B*. *g.* (2)	Hybrid-2 (2)	Hybrid-2 (4)
March 2017	Djémitédouo (N)	*B. t.* (1)	*S*. *h.* (1)	*S*. *h.* (1)
		*B. t.* (1)	Hybrid-1 (1)	*S*. *b.* (1) *+* Hybrid-1 (1)
		*B. t.* (1)	Hybrid-1 (2)	Hybrid-1 (1)
	Korokara (N)	*B. t.* (2)	*S*. *b.* (3)	*S*. *b.* (4)
		*B. t.* (1)	*S*. *b.* (2)	*S*. *b.* (1) + Hybrid-1 (1)
	Noumousso (N)	*B. t.* (1)	*S*. *b.* (2)	*S*. *b.* (2)
	Foroforo (C)	*B. t.* (1)	–	*S*. *b.* (2)
	Kongobo (C)	*B. t.* (1)	*S*. *h.* (2)	*S*. *h.* (2)

Abbreviations: *B. t.*, *Bulinus truncatus*; *B*. *g.*, *B*. *globosus*; *S*. *h.*, *Schistosoma haematobium*; *S*. *b.*, *S*. *bovis*; –, no data obtained; N, North; C, Central

Hybrid profile: Hybrid-1 = *S*. *h.* × *S*. *b.* = *S. b.* mitochondrial *cox*1 and nuclear *S. h* ITS sequence; Hybrid-2 = *S*. *b.* × *S*. *h.* = *S. h.* mitochondrial *cox*1 and mixed *S. h.* and *S. b.* nuclear ITS sequence

### *Bulinus* spp. collections and infection prevalences

A total of 3602 *Bulinus* snails were collected over the three time points in 92 out of the 164 human-water contact sites visited, with 53.4% *B. truncatus*, 39.5% *B. forskalii* and 7.1% *B. globosus*. Stratified by sampling time point, in June 2016, November-December 2016 and March 2017, the numbers of snails sampled were 1128 (56.2% *B. truncatus*, 42.5% *B. forskalii* and 1.3% *B. globosus*), 847 (61.2% *B. forskalii*, 22.8% *B. globosus* and 16.0% *B. truncatus*) and 1627 (70.9% *B. truncatus*, 26.2% *B. forskalii* and 2.9% *B. globosus*), respectively. A total of 2417 surviving *Bulinus* snails were screened for infection, among which 1% (18 *B. truncatus* and 7 *B. globosus*) were found to be shedding mammalian schistosomes (Table 1). Infection prevalence varied by collection time point with estimates of 0.7% (4 *B. truncatus*) (95% CI: 0.2–2.0%) in June 2016, 4% (5 *B. truncatus* and 7 *B. globosus*) (95% CI: 2.0–7.0%) in November-December 2016 and 0.6% (9 *B. truncatus*) (95% CI: 0.2–1.1%) in March 2017 (Table 1 and Additional file 1: Table S1). The molecular identification of the infected snails matched the morphological identification made at the time of collection. The geographical distribution and proportions of each of the snail species collected at sites where positive snails were found are shown in Fig. 1. None of the *B. forskalii* collected in the March 2017 survey shed schistosome cercariae.

### Schistosome cercariae identification

Complete genetic profiles (*cox*1 and ITS) (Table 1) were obtained from schistosome cercariae recovered from each of the 25 infected *Bulinus*; with good quality genetic profiles obtained from 75 individual cercariae. Cercariae with four different genetic profiles were identified: (i) pure species *S. haematobium* (*S. h.* *cox*1 and *S. h.* ITS); (ii) pure species *S. bovis* (*S. b.* *cox*1 and *S. b.* ITS); (iii) hybird *S. haematobium* × *S. bovis* (*S. h.* ×* S. b.*) (*S. b.* *cox*1 and *S. h.* ITS) from now on referred to as hybrid-1; and (iv) hybrid *S. bovis* × *S. haematobium* (*S. b.* × *S. h.*) (*S. h.* *cox*1 and *S. h.* & *S. b.* mixed ITS) from now on referred to as hybrid-2 (Table 1). Figure 1 shows the distribution and proportions of the different *Schistosoma* species and hybrids from the different villages where the positive snails were found. *Cox*1 sequence data accession numbers are: MK333532-MK333538. Four *S. bovis cox*1 haplotypes were identified and one *S. haematobium cox*1 haplotype, which matched the generic mainland (H1) *S. haematobium* [2]. ITS data matched that previously accessioned FJ588857-FJ588862 [14].

### *Bulinus-Schistosoma* infection dynamics

The species and numbers of infected *Bulinus*, together with the species of the schistosomes involved in the infection, from each site and at each time point are shown in Table 1.

Of the 25 infected snails analysed, 44% (13 *B. truncatus*) shed *S. bovis*, 20% (2 *B. truncatus* and 3 *B. globosus*) shed *S. haematobium* and 36% (5 *B. truncatus* and 5 *B. globosus*) shed the hybrids-1 + 2, with no statistical significant difference among these proportions (Fisher’s exact test, *P* > 0.05); however, *S. bovis* infections were clearly more abundant and hybrid-1 is more common than hybrid-2. Schistosome proportions also varied significantly according to the *Bulinus* species involved, with *S. bovis* and the hybrids (1 + 2) being more prevalent in *B. truncatus* and *S. haematobium* and hybrids (1 + 2) being more prevalent in *B. globosus* (Fisher’s exact test, *P* < 0.001). *Schistosoma bovis* was only found infecting *B. truncatus* and *S. haematobium* and the hybrids were found infecting *B. truncatus* and *B. globosus.* No effect of the seasonal time of collection influenced the proportions of the schistosome species (Fisher’s exact test, *P* > 0.05); however, infected *B. globosus* were only found in November-December whereas infected *B. truncatus* were found at all time points.

### Co-infections and cercarial chronobiology

Interestingly, based on our limited number of cercariae analysed, no mixed infections with both pure *S. bovis* and *S. haematobium* were observed in any individual snail (Table 1). However, one *B. globosus* (from Raviart) exhibited a mixed infection with both pure *S. haematobium* and the *S. haematobium* × *S. bovis* hybrid-1, and two *B. truncatus* [from Djémitédouo (*n* = 1) and Korokara (*n* = 1)] were found infected with both pure *S. bovis* and the *S. bovis* × *S. haematobium* hybrid-1.

There was no correlation between the emergence of the different *Schistosoma* species and the time of shedding with *S. haematobium*, *S. bovis* and the *S. haematobium* × *S. bovis* hyrbids shedding both in the morning and the afternoon. However, the only 3 snails (1 *B. globosus* and 2 *B. truncatus*) that were found to have co-infections (detailed above) exhibited differences in the shedding of their schistosome species and/or hybrids at the different times of day. These co-infections may have been missed if shedding had only been performed at a single time point.

### Geographical distribution of the schistosomes identified

Among the 2417 snails tested, 0.52% infected individuals (all *B. truncatus*) were from northern sites, and 0.48% (7 *B. globosus* and 5 *B. truncatus*) were from the central parts of Côte d’Ivoire (Fig. 1). The percentages of snails from the north infected with *S. bovis*, *S. haematobium* and the hybrids-1 + 2 were 3.2%, 0.03% and 0.08%, respectively. Likewise, infection rates in intermediate host snails from central sites were 0.6%, 0.7% and 0.9% with *S. bovis*, *S. haematobium* and the hybrid-1 + 2, respectively. These proportions varied significantly between the northern and central parts of Côte d’Ivoire (Fisher’s exact test, *P* < 0.001), with *S. bovis*-infected *Bulinus* predominantly found in the northern sites, while *S*. *haematobium* and hybrid-1 + 2 infected *Bulinus* snails were mainly obtained from central parts of the study area (Fig. 1). Infected snails were only found in lake environments, apart from one infected snail from a rivulet site in Raviart.

## Discussion

These molecular data from schistosome cercariae from Côte d’Ivoire clearly show the sympatric distribution of *S. haematobium* and *S. bovis* causing human urogenital and veterinary intestinal schistosomiasis, respectively, as previously reported [23, 28], and also confirm the presence of *S. haematobium* × *S. bovis* hybrids. Both *B. globosus* and *B. truncatus* were found infected; *B. truncatus* was predominately infected with *S. bovis* while *B. globosus* (found in two villages in the central regions; Foroforo and Raviart) was only found infected in one village, transmitting *S. haematobium* and the hybrids. In the northern regions, more snails were found infected with *S. bovis* than with *S. haematobium* or hybrids, which is in line with findings from N’Goran [23]. The greater abundance of *S. bovis* in the north compared with central regions may be due to the different frequencies of humans and cattle in these areas and their potential to contaminate the water bodies through urination or defecation. Particularly in the north, the water contact frequency of animals was observed to be more common compared with the human populations. Indeed, in this part of Côte d’Ivoire, where pastoralism is common, some water bodies had been polluted by livestock, and hence, were not frequented by the local human population. Interestingly, none of the *B. forskalii* snails collected were found infected. However, due to the high mortality rate of these snails upon capture and transfer to the laboratory, our data are limited to only one of the survey time points (March 2017), and hence, caution is required for interpreting these observations.

*Schistosoma haematobium* × *S. bovis* hybrids are now known to be common in other parts of West Africa, including Niger [41, 42], Senegal [14, 15] and Mali [43]. Our data widen the extent of this hybridization to Côte d’Ivoire. N’Goran et al. [44] reported the existence of two ‘ecotypes’, based on morphological observations, of *S. haematobium* in Côte d’Ivoire: one that was compatible with *B. truncatus* in the north and the other, which was compatible with *B. globosus* in the south. It is conceivable that these ‘ecotypes’ may have been mixed populations, including hybrids. Our ability to capture, preserve and molecularly characterize larval schistosomes has enhanced knowledge on the epidemiology of these important schistosome species in several endemic countries, highlighting the complexities of their transmission biology. However, the limitations of the molecular markers used does need to be considered. To gain further insights into these inter-species interactions and hybridisation events, it is necessary to start investigating more genomic regions and indeed when possible whole genomes.

The occurrence of these hybrid schistosomes in Côte d’Ivoire may have been influenced by the sympatric water contact behaviour of their respective definitive hosts [14, 15]. Indeed, in the study villages, there are many man-made lakes formed from dam constructions to facilitate multiple agricultural and husbandry activities [13, 22, 23]. Additionally, more than 80% of the contact sites surveyed here, were identified within these types of man-made multi-purpose lakes that are regularly frequented by both humans (for crop irrigation, fishing, domestic tasks, recreation and water provision) and by livestock (for watering) [26, 27].

In some cases, hybrid schistosomes have been shown to exhibit extended host ranges and, enhanced infectivity and virulence [17, 18]. Consequently, it is important to investigate this hybridisation in relation to human transmission and the introduction or spread of infections, such as that observed in Corsica [19]. Both reciprocal hybrids; *S. haematobium* (ITS) × *S. bovis* (*cox*1) (hybrid-1) and mix *S. bovis/S. haematobium* (ITS) × *S. haematobium* (*cox*1) (hybrid-2) were identified, providing evidence for bidirectional introgressive hybridisation, as also previously reported in Senegal [14, 15]. Additionally, the *S. haematobium* (ITS) × *S. bovis* (*cox*1) hybrids-1 were more abundant in the snails than the reciprocal form, which is a common trend [14]. Historical studies, based on chetotaxy and allozymic markers, have also suggested the occurrence of natural *S. bovis* × *S. curassoni* hybrids in northern and central parts of Côte d’Ivoire, which have also been characterized in Niger and Senegal [41, 45]. However, no *S. curassoni* molecular signal was observed in these data [21] and no *Bulinus umbilicatus*, the intermediate host snail of *S. curassoni* was found.

Our data suggest that, although limited due to the small number of cercariae analysed, co-infections within individual snails were only observed when hybrids were involved (*S. bovis* + hybrid or *S. haematobium* + hybrid) but not between pure species e.g. *S. bovis* and *S. haematobium*. This may be due to either fine-scale specific snail-schistosome compatibilities, an acquired resistance mechanism to a second parasitic infection [46, 47], or interspecific competition between parasite larvae in successful mixed infections [48, 49], all of which hybrids may be able to overcome due to their dual inheritance patterns. Additionally, no difference in the chronobiological emergence of the cercariae was observed between *S. bovis*, *S. haematobium* and the hybrids with all three schistosomes shedding in the morning and the afternoon. Additional research is needed to deepen our knowledge of snail resistance, compatibility and susceptibility of these different species and hybrid forms.

## Conclusions

Most of the positive snails found within this study were transmitting *S. bovis* (44%) or *S. haematobium* × *S. bovis* hybrids (36%), while far fewer snails were transmitting *S. haematobium* (20%). The high level of transmission (although focal) of both human urogenital and livestock intestinal schistosomiasis, warrants increased control efforts and surveillance. Our data highlight the necessity to use molecular methods to identify which schistosome species are being transmitted by the different intermediate host snail species in order to refine control and elimination efforts. Our work provides a clearer picture of the focal epidemiology of schistosomiasis and also highlights the possibility of overestimating the level of transmission of human schistosomiasis if animal schistosomes are ignored. Additionally, schistosomes can easily be imported into new areas through the movement of their mammalian hosts, particularly livestock and humans [19, 25, 50]. These factors emphasise the need for accurate cercariae identification during transmission monitoring.

Taken together, we have enhanced knowledge of the epidemiology and transmission dynamics of *S. haematobium* and *S. bovis* in a large part of Côte d’Ivoire and also provided the first conclusive evidence for the transmission of *S. haematobium* × *S. bovis* hybrids in this West African country. This hybridization is clearly widespread in West Africa and the biological characteristics of hybrids, due to heterosis effects, may have a detrimental impact on human health and schistosomiasis control programmes, warranting further research into these inter-species interactions.

## Additional file

**Additional file 1: Table S1.** Snail collection and schistosome infection data. N = North, C = Central. Snail identification is based on shell morphology, except for the positive snails that were molecularly identified.

## Abbreviations

S. h: S. haematobium
S. b: S. bovis
BLAST: Basic Local Alignment Search Tool
*cox*1: Cytochrome *c* oxidase subunit 1
DNA: Deoxyribonucleic acid
GPS: Global positioning system
ITS: Internal transcribed spacer
PCR: Polymerase chain reaction
SCAN: Schistosomiasis Collections at the Natural History Museum
SCORE: Schistosomiasis Consortium for Operational Research and Evaluation
WHO: World Health Organization
Hybrid-1: *S*. *h.* × *S*. *b.* = *S. b.* mitochondrial *cox*1 and nuclear *S. h* ITS sequence
Hybrid-2: *S*. *b.* × *S*. *h.* = *S. h.* mitochondrial *cox*1 and mixed *S. h.* and *S. b.* nuclear ITS sequence
